# The impact of a human IGF-II analog ([Leu^27^]IGF-II) on fetal growth in a mouse model of fetal growth restriction

**DOI:** 10.1152/ajpendo.00379.2015

**Published:** 2015-11-03

**Authors:** Jayne C. Charnock, Mark R. Dilworth, John D. Aplin, Colin P. Sibley, Melissa Westwood, Ian P. Crocker

**Affiliations:** ^1^Maternal and Fetal Health Research Centre, Institute of Human Development, University of Manchester, Manchester, United Kingdom; and; ^2^Maternal and Fetal Health Research Centre, St. Mary's Hospital, Central Manchester Universities National Health Service Foundation Trust, Manchester Academic Health Sciences Centre, Manchester, United Kingdom

**Keywords:** fetal growth restriction, insulin-like growth factor, endothelial nitric oxide synthase knockout, [Leu^27^]insulin-like growth factor II

## Abstract

Enhancing placental insulin-like growth factor (IGF) availability appears to be an attractive strategy for improving outcomes in fetal growth restriction (FGR). Our approach was the novel use of [Leu^27^]IGF-II, a human IGF-II analog that binds the IGF-II clearance receptor IGF-IIR in fetal growth-restricted (FGR) mice. We hypothesized that the impact of [Leu^27^]IGF-II infusion in C57BL/6J (wild-type) and endothelial nitric oxide synthase knockout (eNOS^−/−^; FGR) mice would be to enhance fetal growth and investigated this from mid- to late gestation; 1 mg·kg^−1^·day^−1^ [Leu^27^]IGF-II was delivered via a subcutaneous miniosmotic pump from E12.5 to E18.5. Fetal and placental weights recorded at E18.5 were used to generate frequency distribution curves; fetuses <5th centile were deemed growth restricted. Placentas were harvested for immunohistochemical analysis of the IGF system, and maternal serum was collected for measurement of exogenously administered IGF-II. In WT pregnancies, [Leu^27^]IGF-II treatment halved the number of FGR fetuses, reduced fetal(*P* = 0.028) and placental weight variations (*P* = 0.0032), and increased the numbers of pups close to the mean fetal weight (131 vs. 112 pups within 1 SD). Mixed-model analysis confirmed litter size to be negatively correlated with fetal and placental weight and showed that [Leu^27^]IGF-II preferentially improved fetal weight in the largest litters, as defined by number. Unidirectional ^14C^MeAIB transfer per gram placenta (System A amino acid transporter activity) was inversely correlated with fetal weight in [Leu^27^]IGF-II-treated WT animals (*P* < 0.01). In eNOS^−/−^ mice, [Leu^27^]IGF-II reduced the number of FGR fetuses(1 vs. 5 in the untreated group). The observed reduction in FGR pup numbers in both C57 and eNOS^−/−^ litters suggests the use of this analog as a means of standardizing and rescuing fetal growth, preferentially in the smallest offspring.

fetal growth restriction (FGR) affects 3–10% of babies worldwide and is associated with increased risk of stillbirth, postnatal disability, and impaired motor and cognitive functions. FGR also has lifelong health implications for affected babies, with increased associated risk of hypertension, cardiovascular disease, stroke, and diabetes in adulthood (1, 2, 3, 35). Currently, early delivery is the only treatment for FGR, resulting in further risks of iatrogenic morbidity and mortality (37).

There are several causes of FGR, including maternal undernutrition, fetal chromosomal anomalies, and placental dysfunction, the last of these being the most common cause in Western countries. Placental dysfunction has several different phenotypic characteristics (41): *1*) the placenta often being smaller, with reduced villous area and thickened exchange barrier (31); *2*) reduced vascular complexity and abnormalities in uterine and fetoplacental blood flow (25); *3*) lower activity of nutrient transporters in the syncytiotrophoblast microvillous and basal plasma membranes (36); and *4*) abnormal placental cell turnover, typified by excessive apoptosis and attenuated proliferation (7, 22).

Normal fetal growth is dependent on interplay between fetal, placental, and maternal factors. One family of growth factors, the insulin-like growth factors (IGF-I and IGF-II) and their cell surface receptors (IGF-IR and IGF-IIR), is known to be critical regulators of this growth (17). In humans, circulating total IGF-I in fetal cord blood correlates with birth weight, and mutations in the IGF-I or IGF-IR genes result in severe FGR (18). In mice, ablation of IGF-I or IGF-II genes or elimination of IGF-IR begets severe growth restriction and/or perinatal lethality (30). Using the placental-specific IGF-II-knockout mouse (P0), in which a placental-specific promoter of IGF-II has been deleted, the additional importance of placentally derived IGF-II on placenta and fetal weight has been verified (10). IGF-I and IGF-II are mediators of placental cell turnover, proliferation, survival, and differentiation in vitro and modulate fetal growth in utero by promoting nutrient transfer and placental blood flow (16, 26, 33, 42). Studies in guinea pigs have confirmed the importance of IGF-I in nutrient partitioning from mother to fetus and the role of maternal IGF-II in enhancing placental development and function (38, 40).

Although the roles of IGF-I, IGF-II, and IGF-IR signaling in the human and animal placenta are affirmed, the significance of IGF-IIR in mediating IGF signaling is less well defined. Evidence for signaling through this receptor is accumulating (14, 15), but classically IGF-IIR has been considered a clearance receptor, trafficking excess IGF-II to lysosomes for degradation. This concept is exemplified in IGF-IIR knockout mice, which exhibit fetal and placental overgrowth (45), as a presumed consequence of surplus IGF-II overactivating fetal IGF-IR.

The development of treatments for FGR is dependent on suitable animal models in which they may be tested, and a number of models have been characterized in sheep (4), guinea pig (6, 28), and rat (43). Recent work on gene knockout and transgenic mouse models of pregnancy disease suggests that these may be particularly useful (12). One such model is the endothelial nitric oxide synthase knockout (eNOS^−/−^) (23), which may be considered a more amenable model for FGR. eNOS^−/−^ fetuses are 10% smaller than their wild-type (WT) counterparts, and this is associated with abnormalities in placental transporter activity and dysregulated utero- and fetoplacental blood flow, similar to observations in human FGR (11).

Although systemic administration of IGF-I or -II may be an appealing approach to address placental insufficiency, ubiquitous tissue expression of IGF receptors may cause off-target effects. A possible alternative is to enhance the bioavailability of endogenous IGF already present at the materno-fetal interface. Theoretically, this should be achievable by blocking IGF-IIR. Previously, we have described methods of influencing IGF-II signaling in vitro by siRNA knockdown of IGF-IIR and by the use of an IGF-IIR-specific agonist, [Leu^27^]IGF-II, to block IGF-II removal and optimize its availability (21). In vitro, this approach encourages signaling through IGF-IR, thereby enhancing mitogenesis and cell survival in placental villous explants. A previous in vivo study administering [Leu^27^]IGF-II to normal pregnant guinea pigs at midgestation demonstrated modified placental morphology, enhanced exchange capacity, and, most importantly, significantly increased fetal weight at term (whereas IGF-II alone did not) (39). Here, we hypothesized that the novel treatment of both normal and FGR mice with [Leu^27^]IGF-II would potentially increase IGF-II, therefore enhancing fetal growth in WT litters and improving weights in the growth-restricted fetuses of pregnant eNOS^−/−^ knockouts.

## METHODS

### 

#### Animals and administration of [Leu^27^]IGF-II.

Experiments were performed in accordance with the UK Animals (Scientific Procedures) Act of 1986 under Home Office Licence No. PPL40/3385. The Local Ethical Review Process of the University of Manchester approved all protocols. eNOS^−/−^ were obtained from Jackson Laboratories (strain B6.129P2-Nos3tm1Unc/J). Homozygous eNOS^−/−^ mice were mated, and the presence of a copulation plug was denoted as day 0.5 of pregnancy. C57/BL6J mice, the eNOS^−/−^ background strain, were used as WT mice. Animals had free access to food (Beekay Rat and Mouse Diet; Bantin & Kingman, Hull, UK) and water and were maintained on a 12:12-h light-dark cycle at 21–23°C. On embryonic day (E)12.5, females were anesthetized using isoflurane inhalation and a 100-μl miniosmotic pump (200D; Alzet) surgically inserted subcutaneously in the upper dorsum. Miniosmotic pumps had previously been prepared to deliver vehicle (0.1 M HCl) or 1 mg·kg^−1^·day^−1^ [Leu^27^]IGF-II (human recombinant protein; GroPep) in 0.1 M HCl for 6 days at a flow rate of 0.51 μl/h. [Leu^27^]IGF-II was delivered to 26 C57/BL6J dams (199 fetuses), and 25 C57/BL6J dams (190 fetuses) received vehicle. In a separate experiment, 11 eNOS^−/−^ dams (74 fetuses) were treated with [Leu^27^]IGF-II, and 11 eNOS^−/−^ dams (86 fetuses) were given vehicle. All animals were euthanized on E18.5 by Schedule 1 procedure in accordance with the UK Animals (Scientific Procedures) Act 1986. Fetuses and placentas were blotted and weighed. Placentas were halved and fixed in 4% (vol/vol) neutral-buffered formalin for histological assessment. In animals in which radioactivity had not been used, maternal and fetal serum were collected and frozen in liquid nitrogen.

#### Fetal weight distribution curves.

Fetal weight histograms were constructed as described previously (12), and a nonlinear regression (Gaussian distribution) analysis was performed. The 5th and 95th percentile weights were calculated as (−Z critical value × SD) + mean for the former and (+Z critical value × SD) + mean for the latter, where Z critical value = 1.645 and SD = standard deviation.

#### Unidirectional materno-fetal clearance of ^14^C-MeAIB across the intact placenta.

The clearance across the placenta of ^14^C-methylaminoisobutyric acid (MeAIB), a nonmetabolizable substrate for the placental amino acid transporter system A, was measured across the intact placenta on E18.5 using an adaptation of the method of Flexner and Pohl (1941), as described previously (5). Following infusion of ^14^C-MeAIB, exsanguination of the dam occurred between 1 and 5 min postinfusion. Fetuses were rapidly collected and assessed for total radiolabel accumulation and compared with a maternal plasma ^14^C-MeAIB disappearance curve generated from current and historical in-house data. This was constructed from dams on E18.5 (*n* = 109, group data for C57/BL6J) and fitted to a one-phase exponential decay model (*r*^2^ > 0.6), as described previously (12). ^MeAIB^Kmf (μl·min^−1^·g^−1^ placenta) was calculated as

$$\text{Kmf}=\frac{\text{Nx}}{\text{W}\int_{0}^{x}\text{Cm}\left(t\right)\text{d}t}$$

where Nx = total radiolabel accumulation (disintegrations/min) by the fetus at x min after injection of radiolabel into the maternal vein, W = placental wet weight (g) and equals the time integral of radioisotope concentration in maternal plasma (disintegrations·min^−1^·μl^−1^) from 0 to x min (taken from the maternal plasma ^14^C-MeAIB disappearance curve).

#### Enzyme-linked immunosorbent assays.

ELISAs were performed according to the manufacturers' protocols. Human IGF-II ELISAs to detect [Leu^27^]IGF-II were performed at a 1:10 dilution (detection range 0.02–9 ng/ml, intra- and interassay coefficient of variation = 12.7 and 18.0%, respectively; Mediagnost).

#### Immunohistochemistry.

Freshly harvested placental tissues were fixed in 4% (vol/vol) neutral-buffered formalin at 4°C overnight, washed in phosphate-buffered saline (PBS), embedded in paraffin wax, and cut into 5-μm serial sections. These were microwaved in 0.1 M sodium citrate buffer for antigen retrieval, and then endogenous peroxide activity was blocked by placing the slides in methanol containing 0.4% (vol/vol) HCl and 0.5% (vol/vol) hydrogen peroxide for 30 min. Tissue sections were washed three times in 0.05 M PBS and then incubated with 5% BSA to block any nonspecific binding sites. Sections were incubated with polyclonal rabbit anti-IGF-II (UK 1:100; Abcam, Cambridge), rabbit anti-IGF-IR (UK 1:100; Fisher Scientific-UK, Loughborough, UK), rabbit anti-IGF-IIR (1:100; R & D Systems, Abingdon, UK), or rabbit IgG (minus primary antibodies, for negative controls; DakoCytomation, Ely, UK) overnight at 4°C. Sections were washed (3 × 5 min with PBS) and then incubated with secondary antibody (biotinylated goat anti-rabbit IgG; 1:500 DakoCytomation) for 1 h at room temperature, followed by avidin-peroxidase (5 μg/ml in 0.125 M Tris·HCl, pH 7.6; Sigma-Aldrich, Gillingham, UK) for 30 min at room temperature. Slides were washed in Tris-buffered saline and incubated for 1–5 min with 0.05% (wt/vol) diaminobenzidine and 0.015% (vol/vol) hydrogen peroxide (Sigma-Aldrich). Tissue sections were counterstained with hematoxylin and visualized by light microscopy (BX41; Olympus UK, London, UK).

#### Statistical analysis.

Generalized linear mixed-model analysis with Sidak posttest was used to determine the effect of drug treatment on fetal weight of pups from litters of varying size (*P* < 0.05 was deemed statistically significant). Data were tested for normality following a D'Agostino & Pearson normality test and subjected to unpaired *t*-test or Mann-Whitney *U*-test accordingly. Variance within groups was determined using an *F*-test of equality of variances (*P* < 0.05). Correlations were determined by linear regression analysis. All statistical analyses were carried out using GraphPad Prism 5.03 software.

## RESULTS

### 

#### The IGF-II axis in murine placenta.

**Fig. 1.**
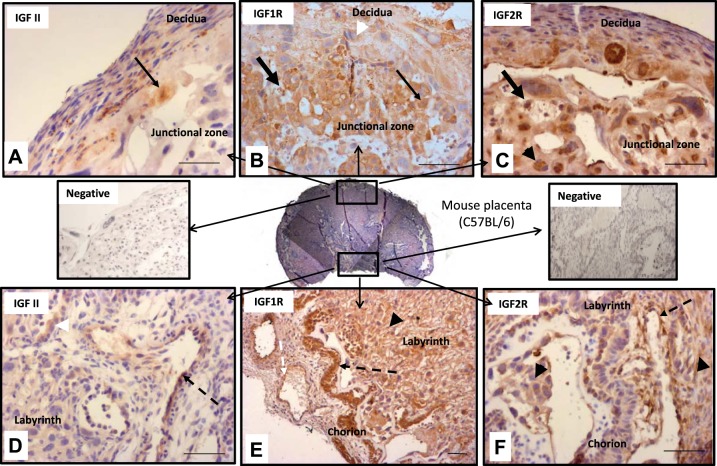
IGF-II axis localization in murine placenta. *A* and *D*: IGF-II was localized throughout the decidua, junctional zone [giant cells (arrow) and glycogen cells; *A*] and labyrinth (trophoblast, arrowhead; vessel endothelium, dashed arrow; *D*). *B* and *E*: IGF-I receptor (IGF-IR) was present throughout the junctional zone (giant cells, arrowhead; trophoblast, thin arrow; glycogen cells, thick arrow; *B*) and labyrinthine trophoblast (arrowhead), with strong expression surrounding vessels and maternal sinuses (dashed arrows) (*E*). *C* and *F*: IGF-IIR displayed a similar localization to IGF-IR, including glycogen cells (thick arrow), trophoblast, (black arrowhead in *C*), labyrinthine trophoblast (black arrowheads in *F*), and endothelium (dashed arrow). Negative staining (minus primary antibody) controls for junctional zone and labyrinth regions also included. Bars, 100 μm.

Previous studies have documented the presence of IGF-II and IGF-IIR mRNA (18, 33, 44), but protein localization has been less well defined. Here, we show that IGF-II is localized throughout the decidua, the junctional zone, including giant cells and glycogen cells, and labyrinth, including trophoblast and vessel endothelium (Fig. 1, *A* and *D*). IGF-IR is expressed by giant cells, trophoblast, and glycogen cells in the junctional zone (Fig. 1*B*) and is also present within labyrinthine trophoblast and the surrounding vessels and maternal sinuses (Fig. 1*E*). IGF-IIR displayed a similar localization to IGF-IR, including glycogen cells, trophoblast, labyrinthine trophoblast, and endothelium (Fig. 1, *C* and *F*).

#### [Leu^27^]IGF-II treatment reduces fetal and placental weight variations in WT mice.

The delivery of [Leu^27^]IGF-II to the maternal circulation was confirmed by analyzing serum from treated and untreated dams using an ELISA specific for human IGF-II; only the samples from WT and eNOS^−/−^ mothers that had received [Leu^27^]IGF-II contained detectable levels of hIGF-II (WT levels: 42.89 ± 11.05 ng/ml, *n* = 4; eNOS^−/−^: 28.47 ± 13.91 ng/ml, *n* = 3; means ± SE).

**Fig. 2.**
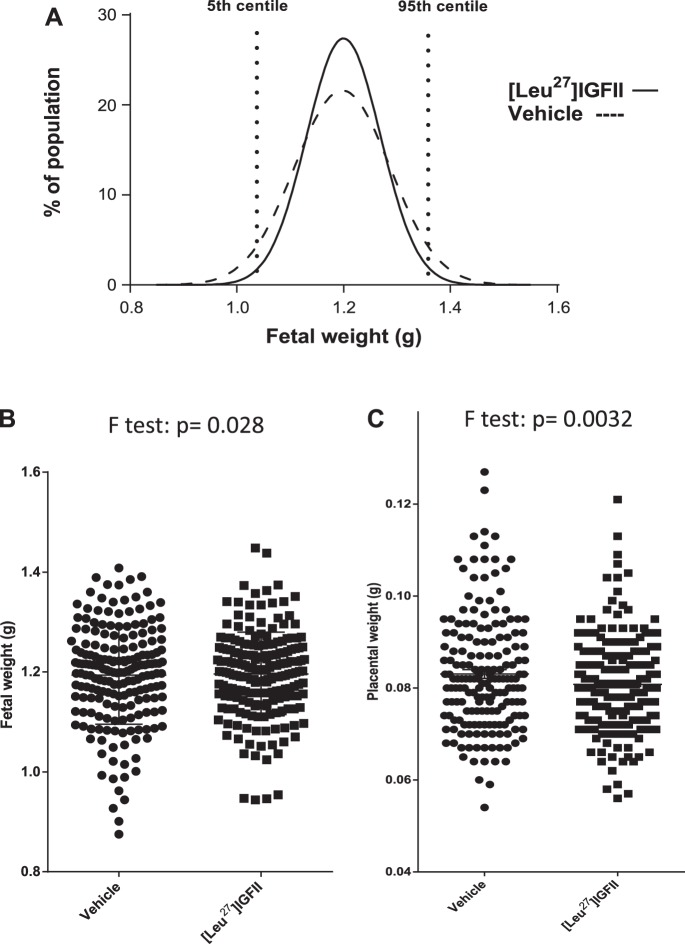
[Leu^27^]IGF-II's effects on fetal weight variation and no. of fetal growth-restricted (FGR) pups in normal mice. Leu^27^: *n* = 26 pregnancies, 199 fetuses; vehicle: *n* = 25, 180 fetuses. *A*: C57 population distribution curves for individual fetal weights of [Leu^27^]IGF-II- (solid line) and vehicle-treated mice (dashed line). Treatment halved the number of pups with <5th centile fetal weights from 6.3 to 2.5% (skewness = 1.14 vs. 1.22) and reduced the number of >95th centile animals from 4.7 to 3%. Individual fetal and placental weights showed reduced variation in treated animals (when tested by *F*-test of equality of variances). *B* and *C*: fetal (*P* = 0.028; *B*) and placental weights (0.0032; *C*).

Treatment of WT mice with [Leu^27^]IGF-II did not alter litter size (7.3 ± 0.4 vs. 7.8 ± 0.3, *n* = 25 and 25, respectively; means ± SE) or number of fetal resorptions (0.52 ± 0.17 vs. 0.69 ± 0.17). Although [Leu^27^]IGF-II did not affect mean fetal weight, population frequency distribution curves revealed that the distribution of weights was narrowed (kurtosis = 0.85 vs. 0.03), with an increase in the number of fetuses close to the mean weight (131 vs. 112 pups within 1 SD) (Fig. 2*A*). Concomitantly, treatment reduced fetal (*P* = 0.028) and placental weight variations (*P* = 0.003), as assessed by *F*-test of equality of variance (Fig. 2, *B* and *C*). Treatment also halved the number of pups with fetal weights below the 5th centile of the control population (skewness = 1.14 vs. 1.22) from 6.3 (*n* = 11) to 2.5% (*n* = 5). The number of pups with fetal weights above the 95th centile also decreased from 4.7 (*n* = 9) to 3.0% (*n* = 6), in line with a normalization effect. Fetal/placental weight ratios, as a measurement of placental efficiency, were unchanged ([Leu^27^]IGF-II-treated ratio = 15.00 ± 0.229, vehicle-treated ratio = 14.94 ± 0.404; means ± SE).

#### [Leu^27^]IGF-II improves fetal weight in large litters in WT mice.

**Fig. 3.**
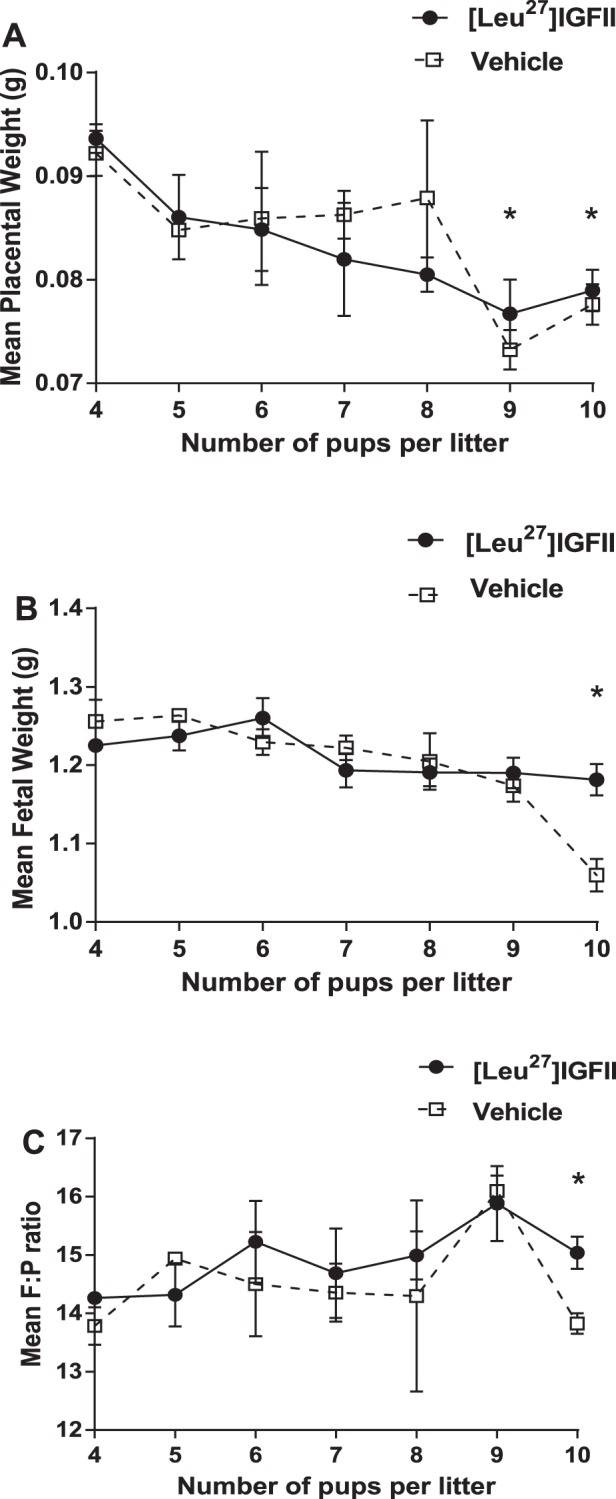
Mean weight/ratio for litters according to size of litter (nos. of pups) and generalized linear mixed-model analysis of [Leu^27^]IGF-II effects. *A*: mean placental weight decreased with increased no. of pups/litter (10 or 9 vs. 4 pups, *P* < 0.05). [Leu^27^]IGF-II had no effect on mean placental weight. *B*: mean fetal weight decreased with increased no. of pups/litter (10 vs. 4 pups, *P* < 0.05). [Leu^27^]IGF-II increased mean fetal weight in largest litters (10 pups). *C*: fetal/placental weight ratio increased in large litters (9 vs. 4, *P* < 0.05). [Leu^27^]IGF-II increased this ratio in the largest litters (10 pups). *P < 0.05.

Generalized linear mixed-model analyses were performed to assess the effects of different variables, including litter size and [Leu^27^]IGF-II treatment, on fetal and placental weights. The mean weight of both placentas and fetuses from litters with the largest number of pups (10) was lower that than of litters with four pups (*P* < 0.05; Fig. 3, *A* and *B*). Treatment with [Leu^27^]IGF-II increased the mean weight of the fetuses (*P* < 0.05), but not placentas, from large litters; however, neither parameter was affected when litter size was small. Consequently, fetal/placental weight ratios were increased only in large litters (*P* < 0.05; Fig. 3*C*).

#### Placental amino acid transport is inversely correlated with fetal weight following maternal [Leu^27^]IGF-II treatment in WT mice.

**Fig. 4.**
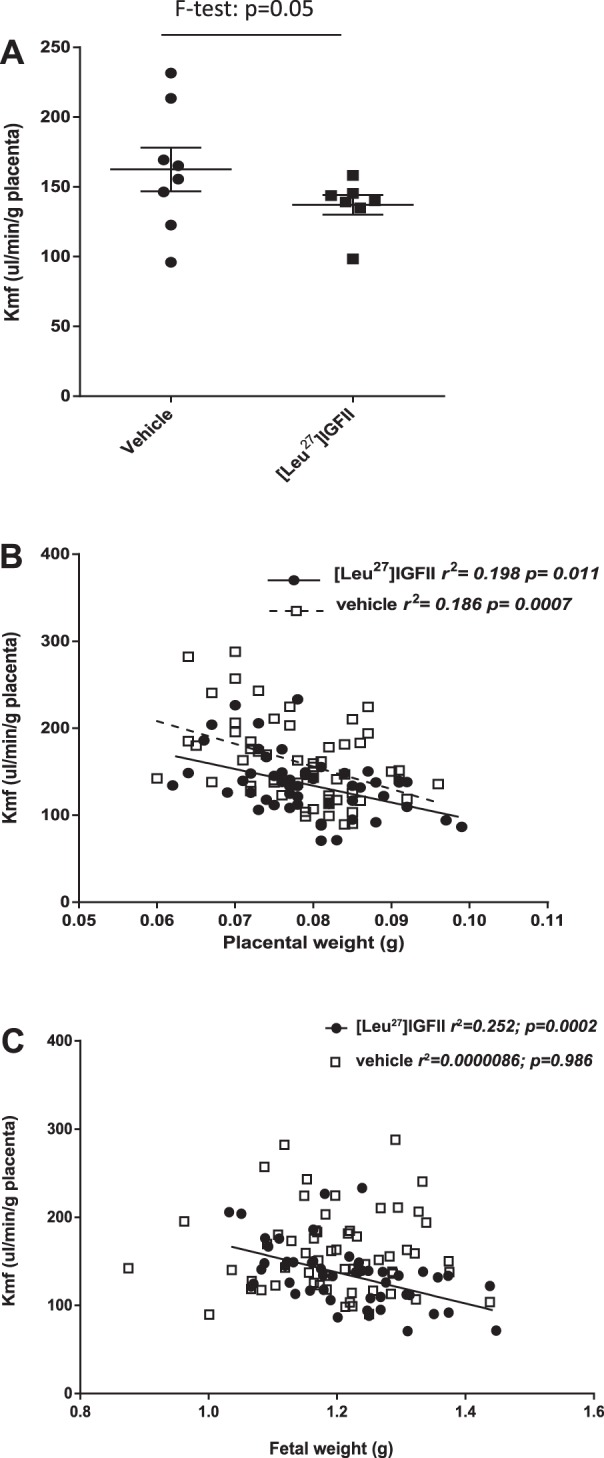
System A amino acid transporter activity in [Leu^27^]IGF-II- (*n* = 7, 51 fetuses) and vehicle-treated (*n* = 8, 60 fetuses) C57 mice. *A*: unidirectional ^14C^MeAIB transfer per gram placenta (kmf) (median ± interquartile range). Variation was significantly reduced in [Leu^27^]IGF-II-treated mice (*P* = 0.05). *B*: transfer (kmf) was inversely correlated with placental weight in both groups ([Leu^27^]IGF-II: *P* = 0.011, *r*^2^ = 0.198; vehicle: *P* = 0.0007, *r*^2^ = 0.186). *C*: transfer (kmf) is inversely correlated with fetal weight in [Leu^27^]IGF-II-treated animals only (*P* < 0.01, line of best fit presented).

To assess the impact of [Leu^27^]IGF-II on placental function, unidirectional materno-fetal clearance of ^14^C-MeAIB (^MeAIB^Kmf) across the placenta, a measure of in vivo system A amino acid transporter activity, was investigated. Activity was not significantly altered following [Leu^27^]IGF-II treatment; however, like the variation in birth weight, there was significantly less variation in clearance between the litters of treated animals (*F*-test *P* = 0.05; Fig. 4*A*). System A amino acid transporter activity was inversely correlated with placental weight in both [Leu^27^]IGF-II- and vehicle-treated animals, with smaller pups exhibiting increased transfer per gram placenta (Fig. 4*B*). An inverse correlation between fetal weight and system A amino acid transporter activity per gram placenta was observed only in the [Leu^27^]IGF-II-treated group (*P* < 0.01; Fig. 4*C*).

#### [Leu^27^]IGF-II treatment reduces the number of growth-restricted fetuses in eNOS^−/−^ mice.

**Fig. 5.**
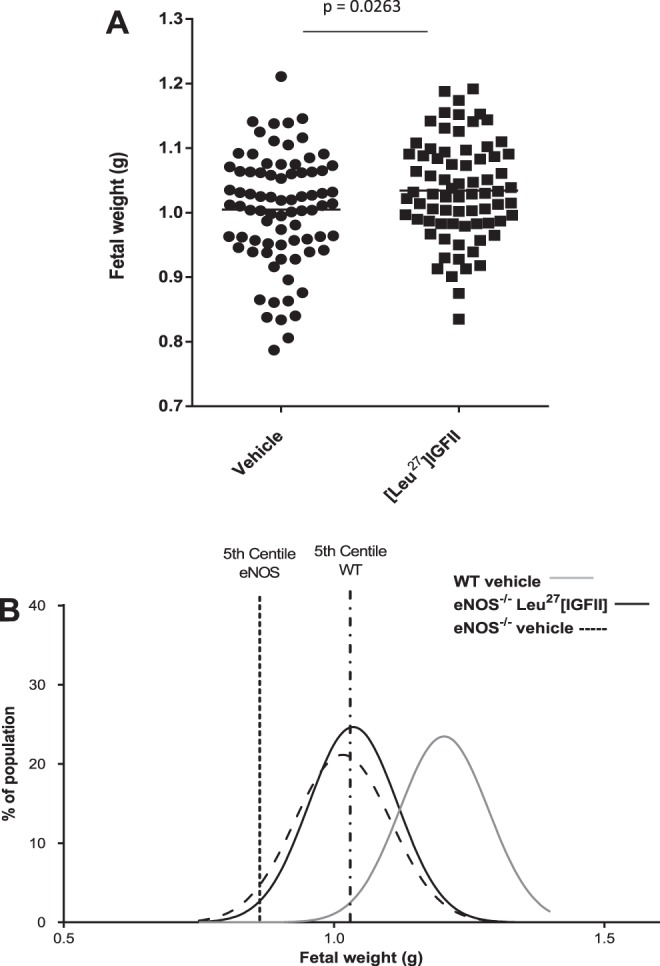
[Leu^27^]IGF-II's effects on fetal weight in endothelial nitric oxide synthase (eNOS) mice (means ± SE). *A*: population distribution curves for individual [Leu^27^]IGF-II-treated eNOS (solid line), vehicle-treated eNOS (dashed line), and vehicle-treated wild-type (WT) (C57; gray line) mice. Treatment reduced the no. of <5th centile eNOS fetuses and the no. of <5th centile fetuses of the C57 population. *B*: individual fetal weights were increased (*P* = 0.0263).

In eNOS^−/−^ mice, [Leu^27^]IGF-II did not affect mean litter fetal or placental weight but significantly increased individual fetal weights (*P* = 0.0263; Fig. 5*A*) and reduced the number of eNOS^−/−^ fetuses below the eNOS^−/−^ population 5th centile by threefold [8.8 (*n* = 5) vs. 2.6% (*n* = 1)]. Treatment also reduced the number below the 5th centile of the WT distribution by 9% (35–44%) (Fig. 5*B*). Placental weights and the variation of fetal weights around the population mean were unchanged ([Leu^27^]IGF-II-treated placental weight = 0.0854 ± 0.0012, vehicle-treated placental weight = 0.0873 ± 0.0015; means ± SE).

## DISCUSSION

The importance of IGF-II signaling in mouse fetal growth is well described, with both total and placental specific IGF-II knockout transgenic animals associated with impaired fetal and placental development (9, 10). Since systemic administration of exogenous IGF-II may have off-target effects, we investigated an alternative approach to optimize fetal growth via constant [Leu^27^]IGF-II infusion in an effort to enhance endogenous IGF-II availability. As reported in the guinea pig (32), we have shown that it is possible to deliver a constant infusion of the IGF-IIR-specific analog [Leu^27^]IGF-II systemically to pregnant mice, without impact on litter size or resorptions. Moreover, with apparent benefits to fetal weight, specifically with regard to the most vulnerable (smallest) pups within the litters in normal and growth-restricted mouse pregnancies, this study holds implications for the further understanding and optimization of fetal growth in utero.

To manipulate the IGF axis at the cellular level, we first confirmed the presence of its constituent components within the mouse placenta. Endogenous IGF-II as well as both IGF receptors, IGF-IR and IGF-IIR, were present throughout the murine placenta, including labyrinthine cells, where nutrient exchange occurs. These findings complement those of others, who have described similar IGF-II mRNA and protein distributions, alongside colocalization of IGF-IR and IGF-IIR principally in mouse trophoblast and vascular endothelium (44). Therefore, theoretically at least, enhancing endogenous IGF-II is an attractive strategy to stimulate placentally driven fetal growth.

We demonstrated that constant subcutaneous infusion of [Leu^27^]IGF-II into WT pregnant mice from midgestation onward reduces the number of fetuses with weights below the 5th centile of the study population. Although fetal sex difference was not considered, we showed [Leu^27^]IGF-II to have its greatest effect in preferentially increasing fetal weights within the smallest, lightest WT fetuses. Because normal animals would be expected to exhibit optimal fetal growth, it could be reasoned that any beneficial effects of treatment might be restricted to fetuses of greater in utero compromise, for example, those under nutritionally stressed circumstances. Therefore, we concluded that pups in large litters compete with varying effectiveness for maternal resources, producing a range of weights in the offspring, and any positive impact on smaller pups can restrict intake by larger pups in the same litter, thus normalizing fetal weights. Within our data, a general inverse relationship between litter size and fetal and placental weights was noted. In the absence of direct changes in placental weight, we envisage this enhancement to be through an exaggeration in functional capacity.

Previous work in WT and P0 knockout mice has shown that an increased placental efficiency, as shown by an increased fetal/placental weight ratio, is associated with increased ^MeAIB^K_mf_ (8, 9, 10). This suggests that increased placental system A amino acid transporter activity at least partially underpins the increased placental efficiency. Therefore, we investigated the relationship between ^MeAIB^K_mf_ and placental and fetal weight in the presence and absence of [Leu^27^]IGF-II by analysis of unidirectional materno-fetal clearance of MeAIB (^MeAIB^Kmf) across placentas as a measure of system A amino acid transport activity. Our data show a strong inverse correlation between ^MeAIB^K_mf_ and placental weight in both the presence and absence of the hormone analog; this is consistent with previous studies linking an increase in placental efficiency with enhanced system A activity. Interestingly, although there was no correlation between ^MeAIB^K_mf_ and fetal weight, which was expected since the increased placental efficiency would tend to drive fetal growth to a constant level across a litter, there was an inverse correlation in the [Leu^27^]IGF-II treated animals. This latter result is intriguing but difficult to decipher. With the relative interplay between placental and fetal weights uncertain, even in WT mice, the uncoupling of this relationship by [Leu^27^]IGF-II by either its effects on amino acid transport or a shift in allocation of resources is a possibility, but one requiring further investigation.

In support of our findings in the mouse, Sferruzzi-Perri et al. (39) have previously demonstrated beneficial effects of [Leu^27^]IGF-II infusion in pregnant guinea pigs from midgestation onward. Resulting increases in fetal weight were attributed to enhanced placental functional activities, with increased system A activity and glucose transfer per gram of placenta. Consistent with the concept that [Leu^27^]IGF-II can normalize (and arguably optimise) fetal growth, we demonstrated a reduction in litter variability in system A transport in the mouse, mirroring its impact on placental and fetal weights and potentially explaining it.

In light of improved fetal weights in constitutively small offspring of WT mice, we investigated for the first time the effects of maternal [Leu^27^]IGF-II infusion in a pregnant mouse model of FGR (eNOS^−/−^). [Leu^27^]IGF-II increased eNOS^−/−^ fetal weights, reducing numbers below the 5th centile, akin to that of wild-type mice. Although [Leu^27^]IGF-II failed to restore mean fetal weights to WT levels, the clinical relevance of such an outcome should not be overlooked, as preferential growth in the smallest offspring could increase fetal weights above critical centiles for both viability and in utero programming, thereby having a significant impact on fetal, neonatal, and longer-term well being in humans. In defining mechanisms, it is suggested that [Leu^27^]IGF-II, like IGF-II, may promote uterine angiogenesis and vascular remodelling (24, 39), improving blood flow and nutrient delivery. Although not assessed in this study, the eNOS^−/−^ mouse exhibits altered uterine vascular function, which may be improved through [Leu^27^]IGF-II exposure (41).

There are a number of signaling mechanisms by which [Leu^27^]IGF-II may elicit either maternal or fetal effects. Previous work by our group (21, 27), using human placental explants, indicates that likely saturation of IGF-IIR by [Leu^27^]IGF-II may enhance endogenous IGF-II bioavailability and increase signaling through IGF-IR, augmenting its well-documented growth-promoting properties. Within that study, decreased IGF-IIR expression increased an IGF-II-mediated impact on mitosis and placental cell survival. Alongside these observations and the knowledge that IGF-IIR knockout mice elicit fetal overgrowth, these findings imply a role for IGF-IIR blockade in stimulating tissue growth via increasing IGF-II bioavailability and in turn IGF-IR activation (29). Furthermore, the effects on maternal adiposity described by Sferruzzi-Perri et al. (39), which were similar to those observed previously following IGF-I (but not IGF-II) treatment, also support the concept of endogenous IGF-II displacement to enhance its interaction with IGF-IR. In addition, IGF-IIR may itself have direct signaling capabilities, rather than acting exclusively as a clearance receptor (21). Further in vitro studies have demonstrated the capacity of [Leu^27^]IGF-II signaling via IGF-IIR to stimulate activation of MARK3/1, resulting in trophoblast migration and cell survival (32). Therefore, although signaling pathways within pregnant mice remain uncertain, both direct and indirect IGF receptor interactions may be balanced within the placenta to ensure appropriate placental and fetal growth.

In summary, we have defined a reduction in FGR pups in WT and eNOS^−/−^ litters exposed to a maternal exogenous analog of IGF-II. Under normal circumstances, we propose that the IGF axis is already optimized for any given fetus, and as such [Leu^27^]IGF-II-treatment has little or no positive effect on normal mice, as IGF-IIR is saturated. By contrast, in compromised, inadequately growing fetuses (i.e., smaller pups where IGF-II is potentially restricted), [Leu^27^]IGF-II acts to promote availability of endogenous IGF-II, restoring placental sufficiency and standardizing fetal growth, rescuing the FGR phenotype.

## GRANTS

We acknowledge the support of the Castang Foundation in funding this project, as well as an Medical Research Council (MRC) Programme Grant (Sibley G0802770/92495). M. R. Dilworth is supported by a Career Development Fellowship award from the MRC (Grant No. MR/K024442/1).

## DISCLOSURES

The authors have nothing to disclose.

## AUTHOR CONTRIBUTIONS

J.C.C., J.D.A., C.P.S., M.W., and I.P.C. conception and design of research; J.C.C. and I.P.C. performed experiments; J.C.C., M.R.D., J.D.A., and I.P.C. analyzed data; J.C.C., J.D.A., C.P.S., M.W., and I.P.C. interpreted results of experiments; J.C.C., M.R.D., and I.P.C. prepared figures; J.C.C. and I.P.C. drafted manuscript; J.C.C., J.D.A., C.P.S., M.W., and I.P.C. approved final version of manuscript; M.R.D., J.D.A., C.P.S., M.W., and I.P.C. edited and revised manuscript.
